# Functional neuroimaging biomarkers of anhedonia response to escitalopram plus adjunct aripiprazole treatment for major depressive disorder

**DOI:** 10.1192/bjo.2023.588

**Published:** 2024-01-05

**Authors:** Sophie R. Vaccarino, Shijing Wang, Sakina J. Rizvi, Wendy Lou, Stefanie Hassel, Glenda M. MacQueen, Keith Ho, Benicio N. Frey, Raymond W. Lam, Roumen V. Milev, Susan Rotzinger, Arun V. Ravindran, Stephen C. Strother, Sidney H. Kennedy

**Affiliations:** Institute of Medical Science, University of Toronto, Canada; Centre for Depression and Suicide Studies, Unity Health Toronto, Canada; and Cumming School of Medicine, University of Calgary, Canada; Institute of Medical Science, University of Toronto, Canada; and Centre for Depression and Suicide Studies, Unity Health Toronto, Canada; Institute of Medical Science, University of Toronto, Canada; Centre for Depression and Suicide Studies, Unity Health Toronto, Canada; Department of Psychiatry, University of Toronto, Canada; Department of Psychiatry, Unity Health Toronto, Canada; and Li Ka Shing Knowledge Institute, Unity Health Toronto, Canada; Dalla Lana School of Public Health, University of Toronto, Canada; and Department of Biostatistics, University of Toronto, Canada; Cumming School of Medicine, University of Calgary, Canada; and Department of Psychiatry, University of Calgary, Canada; Centre for Depression and Suicide Studies, Unity Health Toronto, Canada; Department of Psychiatry, Unity Health Toronto, Canada; and Li Ka Shing Knowledge Institute, Unity Health Toronto, Canada; Department of Psychiatry and Behavioural Neurosciences, McMaster University, Canada; Department of Psychiatry, University of British Columbia, Canada; Department of Psychiatry, Providence Care, Queen's University, Canada; Centre for Depression and Suicide Studies, Unity Health Toronto, Canada; Department of Psychiatry, University of Toronto, Canada; Institute of Medical Science, University of Toronto, Canada; Rotman Research Institute, Baycrest Centre, Canada; and Department of Medical Biophysics, University of Toronto, Canada; Institute of Medical Science, University of Toronto, Canada; Centre for Depression and Suicide Studies, Unity Health Toronto, Canada; Department of Psychiatry, University of Toronto, Canada; Department of Psychiatry, Unity Health Toronto, Canada; Li Ka Shing Knowledge Institute, Unity Health Toronto, Canada; and Krembil Research Institute, University Health Network, Toronto, Canada

**Keywords:** Antidepressants, antipsychotics, depressive disorders, other imaging, anhedonia

## Abstract

**Background:**

Identifying neuroimaging biomarkers of antidepressant response may help guide treatment decisions and advance precision medicine.

**Aims:**

To examine the relationship between anhedonia and functional neurocircuitry in key reward processing brain regions in people with major depressive disorder receiving aripiprazole adjunct therapy with escitalopram.

**Method:**

Data were collected as part of the CAN-BIND-1 study. Participants experiencing a current major depressive episode received escitalopram for 8 weeks; escitalopram non-responders received adjunct aripiprazole for an additional 8 weeks. Functional magnetic resonance imaging (on weeks 0 and 8) and clinical assessment of anhedonia (on weeks 0, 8 and 16) were completed. Seed-based correlational analysis was employed to examine the relationship between baseline resting-state functional connectivity (rsFC), using the nucleus accumbens (NAc) and anterior cingulate cortex (ACC) as key regions of interest, and change in anhedonia severity after adjunct aripiprazole.

**Results:**

Anhedonia severity significantly improved after treatment with adjunct aripiprazole.

There was a positive correlation between anhedonia improvement and rsFC between the ACC and posterior cingulate cortex, ACC and posterior praecuneus, and NAc and posterior praecuneus. There was a negative correlation between anhedonia improvement and rsFC between the ACC and anterior praecuneus and NAc and anterior praecuneus.

**Conclusions:**

Eight weeks of aripiprazole, adjunct to escitalopram, was associated with improved anhedonia symptoms. Changes in functional connectivity between key reward regions were associated with anhedonia improvement, suggesting aripiprazole may be an effective treatment for individuals experiencing reward-related deficits. Future studies are required to replicate our findings and explore their generalisability, using other agents with partial dopamine (D2) agonism and/or serotonin (5-HT2A) antagonism.

Major depressive disorder (MDD) is characterised by ‘depressed mood’ and/or anhedonia. Anhedonia is classically defined as a ‘diminished interest or pleasure in response stimuli that were previously perceived as rewarding during a pre-morbid state’,^1^ and is recognised as a core feature of MDD in both the DSM and the World Health Organization's ICD. Up to 70% of individuals with MDD present with anhedonia symptoms,^2^ confirming anhedonia as a major feature of MDD. Individuals with anhedonia experience greater overall depressive severity^3^ and decreased antidepressant treatment response.^4,5^ In fact, anhedonia may be one of the most persistent symptoms of MDD, despite clinical remission from a major depressive episode (MDE): approximately 25% of individuals in remission continue to have residual symptoms of anhedonia.^6^

## Anhedonia treatment in MDD

Currently, serotonergic antidepressants are first-line treatments for MDD. However, there is mixed evidence regarding the efficacy of selective serotonin reuptake inhibitors (SSRIs) and serotonin-norepinephrine reuptake inhibitors (SNRIs) in improving anhedonia in individuals with MDD. Several studies of pharmacological interventions for anhedonia in MDD concluded that, although there was an improvement in anhedonia after treatment with an SSRI or SNRI, this improvement was significantly less than observed with other antidepressant agents, such as agomelatine.^7^ A possible explanation for the limited anti-anhedonia effects of SSRIs is the fact that anhedonia is primarily associated with dopaminergic and opioidergic dysfunction, rather than serotonergic.^8^ Further, SSRI-associated emotional blunting is a relatively common adverse event among patients receiving SSRIs;^9^ therefore, SSRIs may sometimes exacerbate anhedonia.

The shortcomings of SSRIs in improving anhedonia present the need for other agents that specifically address the underlying pathophysiology of anhedonia. Dopamine is among the most frequently implicated neurotransmitters associated with reward processing and anhedonia,^8^ and so it stands to reason that medications with dopaminergic properties may be effective in treating anhedonia. This is supported by reports on a number of medications with dopaminergic actions – including agomelatine, bupropion, pramipexole and psychostimulants – which have been associated with an improvement in anhedonia among individuals with MDD^7^ and other psychiatric/neurological disorders (e.g. Parkinson's disease).^10^ Aripiprazole, an atypical antipsychotic that has dopamine D2 receptor agonist properties and is an approved adjunct treatment for MDD, may also reduce symptoms of anhedonia.

## The brain's reward circuitry

Advances in applied functional brain imaging to examine neural reward circuits have enhanced our understanding of reward deficits and various aspects of anhedonia, which involve communication between several key brain regions, including the ventral tegmental area (VTA), nucleus accumbens (NAc), ventral pallidum and anterior cingulate cortex (ACC). Theoretically, dysregulation in any of these regions may cause anhedonia. Among brain networks, the salience network is most implicated in reward processing: it selectively enhances salient reward stimuli required for higher cognitive processing from the flow of incoming stimuli with which humans are constantly presented.^11,12^ Several brain regions are implicated in the salience network: although the anterior insula and dorsal ACC act as the main hubs of the salience network, there is also significant contribution from the amygdala, ventral striatum, thalamus, hypothalamus, substantia nigra pars compacta and VTA.^11,12^

## The current study

The purpose of the current study is to examine the relationship between anhedonia and functional neurocircuitry in key reward processing brain regions in an MDD population treated with the partial D2 agonist aripiprazole as an adjunct to the SSRI escitalopram. Data were collected as part of the first Canadian Biomarker Integration Network in Depression (CAN-BIND-1) study,^13^ in which participants with MDD received escitalopram for 8 weeks. Non-responders to escitalopram were augmented with aripiprazole for a further 8 weeks, whereas responders continued 8 additional weeks of escitalopram only. Neuroimaging and clinical data were collected throughout the 16-week study. The group who received aripiprazole as an adjunct is the population of interest for this report. Our primary objective was to identify baseline and week 8 resting-state functional magnetic resonance imaging (fMRI) biomarkers associated with anhedonia improvement following adjunct aripiprazole treatment. Over the past several years, fMRI has been increasingly used to explore predictors of treatment response in populations with depression (e.g. for treatment with escitalopram, sertraline and pramipexole);^14–17^ however, to the best of our knowledge, this is the first study to explore resting-state fMRI predictors of anhedonia response to aripiprazole in a population with MDD.

## Method

Clinical and neuroimaging data were obtained from the first CAN-BIND trial, CAN-BIND-1 (Clinicaltrials.gov identifier: NCT01655706).^13,18^ CAN-BIND is a Canada-wide research programme that aims to identify biomarkers of antidepressant treatment response.^13,18^ From August 2013 to December 2016, 211 participants meeting the criteria for MDD, who were experiencing a current MDE, were recruited from six Canadian sites: University Health Network (Toronto), Centre for Addiction and Mental Health (Toronto), McMaster University (Hamilton), Queen's University (Kingston), University of Calgary and University of British Columbia (Vancouver). The authors assert that all procedures contributing to this work comply with the ethical standards of the relevant national and institutional committees on human experimentation and with the Helsinki Declaration of 1975, as revised in 2008. All procedures involving human patients were approved by research ethics boards at each study site. This work was previously published by the first author (S.R.V.) as part of a Master's thesis through the Institute of Medical Science, University of Toronto.^19^

### Participants

Detailed inclusion/exclusion criteria, clinical outcomes and sample size calculations are described elsewhere.^13,18^ In summary, eligible participants must have met criteria for an MDE for ≥3 months (based on DSM-IV-TR criteria, as confirmed by the Mini-International Neuropsychiatric Interview), were between the ages of 18 and 60 years, and had been free of psychotropic medications for at least five half-lives. Major exclusion criteria included diagnosis of another primary psychiatric disorder or neurological condition; current psychosis, substance use disorder or high suicide risk; failure of four or more adequate antidepressant trials; and previous failure or intolerance to escitalopram and/or aripiprazole. All participants provided written informed consent after receiving a complete description of the study.

### Intervention

Study participants received open-label escitalopram (10–20 mg/day) for 8 weeks, after which escitalopram ‘non-responders’ (defined as <50% reduction in Montgomery–Åsberg Depression Rating Scale (MADRS) score from start of treatment to week 8) received adjunct aripiprazole (2–10 mg/day) for an additional 8 weeks. Dosing was at the discretion of the individual clinician.

### Primary outcome measures

The primary outcome was anhedonia severity, as measured with the Dimensional Anhedonia Rating Scale (DARS).^4^ This 17-item self-report scale measures four dimensions of anhedonia – desire, motivation, effort and consummatory pleasure – with lower scores reflecting greater anhedonia.^4^ Participants are asked to choose two or three of their favourite activities/experiences to act as prompts for each of four categories: pastimes/hobbies, food/drinks, social activities and sensory experiencing. There were no limitations on which prompts a participant could choose, and they were not required to use the same prompts across sessions. The DARS displays good reliability and divergent validity.^4^ Improvement in anhedonia was measured as percentage change in DARS score, with a higher percentage equating to greater improvement. The DARS was completed by participants at baseline, week 8 and week 16.

Two analyses were completed with functional neuroimaging statistical analysis software (methods described below). First, resting-state fMRI predictors of DARS score change in the adjunct aripiprazole group were analysed from week 8 (aripiprazole baseline) to week 16. Second, resting-state fMRI predictors of DARS score change among the adjunct aripiprazole group were analysed from baseline to week 16.

### Neuroimaging acquisition and processing

Magnetic resonance imaging (MRI) data were collected at baseline and week 8, using 3.0 T MRI scanners. Each scan lasted 10 min and participants focused on a fixation cross projected in the MRI machine during the scan. A whole-brain T2*-sensitive blood oxygenation level dependent echo-planar imaging series was used with the following parameters: 2000 ms repetition time, 30 ms echo time and voxel dimensions of 4 × 4 × 4 mm. Further details of the MRI acquisition protocol for this and other CAN-BIND imaging projects are available elsewhere.^20^ Neuroimaging data were preprocessed with the Optimization of Preprocessing Pipelines for Neuroimaging-fMRI (OPPNI-fMRI) pipeline^21,22^ via Analysis of Functional Neuroimaging (AFNI) software (https://afni.nimh.nih.gov/pub/dist/doc/htmldoc).^23,24^

### Statistical analysis

Seed-based correlational analysis was employed to examine the relationship between resting-state functional connectivity (rsFC) of two regions of interest – NAc and ACC – and change in DARS score. These regions of interest, or ‘seeds’, were chosen because they are strongly and consistently implicated in reward processing and related to anhedonia severity in several previous studies.^25–27^ For the standardised functional image of each patient, a region of interest ‘mask’ was applied to extract the BOLD time series. A whole-brain, seed-based connectivity map was then calculated for each participant, which shows the strength of rsFC between the region of interest and all other voxels in the brain. These maps were subsequently used in higher-level analyses, applying a mass univariate approach, to investigate patterns across participants.^28^ Region of interest masks were created with the Harvard-Oxford Subcortical Structural Atlas for NAc^29^ and the Talairach Atlas for ACC.^30–32^

To investigate the relationship between rsFC and percentage change in DARS score, a general linear model (GLM) framework was applied: the main explanatory variable was change in DARS score, and age and gender were entered to control for confounding. To identify voxel clusters where this relationship was statistically significant, cluster thresholding was used (*Z*-statistic threshold of 3.1, cluster *P*-value threshold of 0.05). Several anatomical atlases were used to identify the Montreal Neurological Institute (MNI) 152 coordinates of the local *Z*-statistic maximum(s) at each cluster.^29,33–37^ This analysis was repeated with MADRS score as an additional explanatory variable to establish whether significant findings were independent of depressive severity. The FMRIB Software Library (FSL) FEAT package was used for this analysis, which also corrects for multiple comparisons by using nonparametric permutation inference (FEAT version 6.00 for macOS, FMRIB, Oxford, UK; see www.fmrib.ox.ac.uk/fsl).^28,38^

Independent component analysis (ICA) was applied to investigate the relationship between rsFC in the salience network and change in DARS score. ICA decomposition was performed with a temporal concatenation approach, via the FSL melodic command line.^39–41^ Twenty components were extracted, employing the methodology of Iraji et al, who used resting-state fMRI data collected from 309 individuals to identify 12 resting-state networks.^42^ To investigate the relationship between rsFC in these resting-state networks and change in DARS score, a GLM framework was used, and age and gender were entered to control for confounding. Dual regression analysis was performed with this GLM matrix, to compare resting-state network maps across participants.^43,44^ Using the FSL ‘randomize’ tool, nonparametric permutation inference with 5000 permutations was completed to correct for multiple comparisons across individuals.^45^ The salience network was identified from the independent components generated, using the map from Iraji et al for guidance.^42^ Both *t*-statistic and *P*-values for each voxel were generated, and *P*-values <0.05 represented salience network regions where rsFC and change in DARS score were significantly correlated. This analysis was repeated with MADRS score as an additional explanatory variable to establish whether findings were independent of depressive severity.

All analyses were also completed for participants who received escitalopram monotherapy for the duration of the study, for comparison purposes. These methods and results are presented in Supplementary Material available at https://doi.org/10.1192/bjo.2023.588.

## Results

Overall, 211 participants were enrolled in the CAN-BIND-1 study (from August 2013 to December 2016). Non-responders to escitalopram (*n* = 95) were eligible to receive adjunct aripiprazole for an additional 8 weeks; of these participants, 69 had complete clinical and neuroimaging data to identify baseline neuroimaging predictors of week 16 response, and 71 participants had complete data to identify week 8 predictors of week 16 response. There were no significant differences in demographic or baseline clinical characteristics between those included and excluded from the subsequent analyses.

There were no differences in demographics or psychiatric history between escitalopram responders and non-responders, although non-responders had received a greater number of previous antidepressant trials than responders (*t* = −2.67, *P* < 0.01). Also, there were no significant differences in baseline MADRS or DARS scores between the two groups.

Among those receiving adjunct aripiprazole, there was a significant improvement in DARS score after 8 weeks of aripiprazole treatment (*t* = −3.48, *P* < 0.001). This improvement was independent of change in MADRS score (Pillai's Trace: 0.315, *P* < 0.001).

Demographic and clinical data are presented in Table 1.

**Table 1:** Baseline demographics of study population

Variable	Study population (*N* = 95)
Age, years, mean (s.d.)	35.8 (13.2)
Education, years, mean (s.d.)	16.9 (2.1)
Female, % (*n*)	60% (57)
Employed, % (*n*)	67% (58)
Never married, % (*n*)	53% (50)
Ethnicity, % (*n*)	
White	69% (66)
Asian	16% (15)
Latin/Hispanic	4% (4)
Black	1% (1)
Mixed	7% (7)
Other	2% (2)
Age at MDD onset, years, mean (s.d.)	21.9 (11.1)
Number of previous MDEs, mean (s.d.)	2.7 (3.4)
Current MDE duration, months, mean (s.d.)	30.5 (37.6)
Number of previous treatments, mean (s.d.)	0.72 (0.91)
Family history of psychiatric illness, % (*n*)	75% (71)
Mean MADRS score (s.d.)	30.5 (5.5)
Mean DARS score (s.d.)	
Baseline	32.3 (15.1)
Week 8	32.6 (14.0)
Week 16	41.2 (17.8)
Change in DARS score,^a^ mean (s.d.)	
Baseline to week 8	1.28 (1.09)
Baseline to week 16	1.62 (1.56)
Week 8 to week 16	1.39 (0.61)

MDD, major depressive disorder; MDE, major depressive episode; MADRS, Montgomery–Åsberg Depression Rating Scale; DARS, Dimensional Anhedonia Rating Scale.

a. Expressed as a fraction, final value divided by initial.

### Seed-based correlation analysis: rsFC association with anhedonia change (week 8 to week 16)

There was a positive correlation between change in DARS score from week 8 to week 16 and week 8 rsFC between the ACC (which was used as the seed) and the posterior cingulate cortex (PCC) (*P* = 0.03), as well as the bilateral ventral-posterior praecuneus (*P* = 0.01) at week 8. Change in DARS score was also negatively correlated with week 8 rsFC between the ACC and right dorsal-anterior praecuneus (*P* = 0.03), middle frontal gyrus (left: *P* = 0.004; right: *P* = 0.02) and left superior frontal gyrus (*P* = 0.004).

**Fig. 1:**
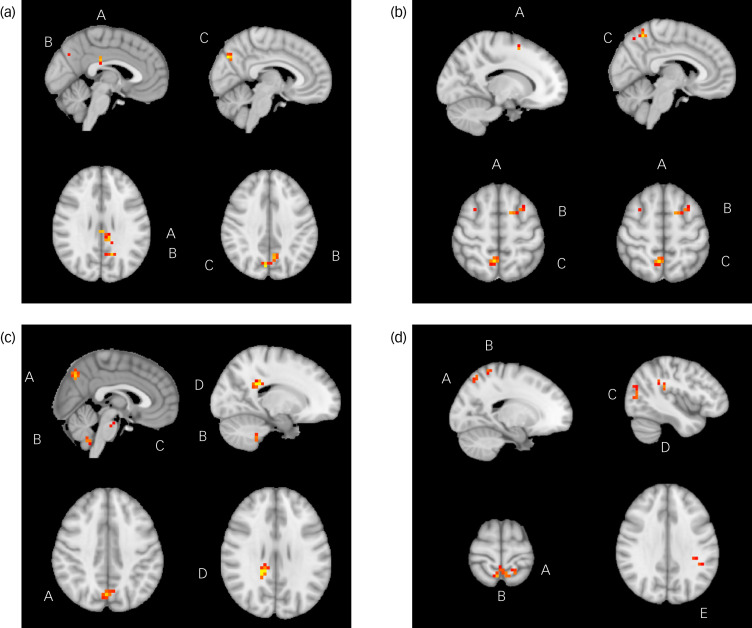
Significant relationships between change in Dimensional Anhedonia Rating Scale (DARS) score and resting-state functional connectivity (rsFC), week 8 to week 16. (a) There were significant positive relationships between change in DARS score and rsFC between the anterior cingulate cortex and posterior cingulate cortex (A), left praecuneus (B) and right praecuneus (C). (b) There were significant positive relationships between change in DARS score and rsFC between the between the anterior cingulate cortex and superior frontal gyrus (A), middle frontal gyrus (B) and right praecuneus (C). (c) There were significant positive relationships between change in DARS score and rsFC between the nucleus accumbens and praecuneus (A), cerebellum lobule IX (B), pons (C) and splenium of corpus collosum (D). (d) There were significant positive relationships between change in DARS score and rsFC between the nucleus accumbens and superior parietal gyrus (A), praecuneus (B), middle occipital gyrus (C), parietal operculum (D) and supramarginal gyrus (E).

Change in DARS score from week 8 to week 16 was also positively correlated with week 8 rsFC between the NAc (which was used as the seed) and bilateral ventral-posterior praecuneus (*P* = 0.001), cerebellum lobule IX (*P* < 0.001), pons (*P* = 0.01) and splenium of the corpus collosum (*P* = 0.03). Change in DARS score was negatively correlated with week 8 rsFC between the NAc and left supramarginal gyrus (*P* = 0.02), parietal operculum (*P* = 0.02), middle occipital gyrus (left: *P* = 0.004; right: *P* = 0.01), superior parietal gyrus (*P* < 0.001) and bilateral dorsal-anterior praecuneus (*P* < 0.001). All results are presented in Table 2, and presented visually in Fig. 1.

**Table 2:** MNI152 coordinates of *Z*-maxima for association between DARS score change and rsFC, week 8 to week 16^a^

Cluster	Number of voxels	*Z*-maxima	MNI152 coordinates (*x*, *y*, *z*)	Brain regions	*P*-value
Positive correlation, rsFC in ACC					
1	17	3.80	(−2, −26, 28), (−10, −34, 28)	Posterior cingulate gyrus	0.029
2	23	3.80	(−6, −62, 36), (−14, −58, 28)	Left praecuneus	0.007
		3.98	(6, −74, 36)	Right praecuneus	
Negative correlation, rsFC in ACC					
1	17	4.30	(10, −66, 52), (6, −54, 56)	Right praecuneus	0.029
2	18	4.41	(30, 14, 48)	Right MFG	0.022
3	25	3.92	(−22, 2, 72), (−14, 6, 56)	Left SFG	0.004
		4.25	(−30, 10, 60)	Left MFG	
Positive correlation, rsFC in NAc					
1	16	4.62	(18, −42, 28)	Splenium of corpus collosum	0.027
2	19	3.40	(−14, −18, −28)	Brainstem	0.012
		4.20	(−2, −26, −28), (10, −30, −28)	Pons	
3	28	4.34	(2, −70, 40)	Praecuneus	0.001
4	35	3.88	(6, −42, −40), (18, −42, −44), (2, −54, −48)	Cerebellum lobule IX	<0.001
		4.03	(−2, −50, −56)	Brain-stem	
Negative correlation, rsFC in NAc					
1	17	3.64	(−42, −42, 32)	Left SMG	0.021
		3.66	(−46, −46, 24)	Left SMG	
		3.97	(−42, −34, 24)	Parietal operculum	
2	19	4.69	(34, −78, 8)	Right MOG	0.012
3	25	3.31	(−38, −74, 24)	Left MOG	0.003
		4.65	(−38, −74, 8)	Occipital cortex	
4	53	3.74	(−22, −66, 48)	Superior parietal gyrus	<0.001
		4.28	(−18, −66, 60), (−18, −50, 68), (−6, −58, 64), (−6, −50, 72)	Left praecuneus	
		4.19	(6, −54, 68)	Right praecuneus	

MNI, Montreal Neurological Institute; DARS, Dimensional Anhedonia Rating Scale; rsFC, resting-state functional connectivity; ACC, anterior cingulate cortex; MFG, middle frontal gyrus; SFG, superior frontal gyrus; NAc, nucleus accumbens; SMG, supramarginal gyrus; MOG, middle occipital gyrus.

a. Controlled for Montgomery–Åsberg Depression Rating Scale score, gender and age.

### Seed-based correlation analysis: rsFC association with anhedonia change (week 0 to week 16)

Change in DARS score from baseline to week 16 was positively correlated with baseline rsFC between the NAc and the supplementary motor area (*P* = 0.001), precentral gyrus (*P* = 0.001), and anterior and posterior cingulate cortices (*P* = 0.001) when controlling for both age and gender; and age, gender and MADRS score. Additionally, when controlling for age, gender and MADRS score (but neither age nor gender alone), change in DARS score was positively associated with baseline rsFC between the NAc and Heschl's gyrus (*P* = 0.001), planum temporale (*P* = 0.001), insula (*P* = 0.001) and central opercular cortex. All results are presented in Table 3, and presented visually in Fig. 1.

**Table 3:** MNI152 coordinates of *Z*-maxima for association between DARS score change and rsFC, baseline to week 16^a^

Cluster	Number of voxels	*Z*-maxima	MNI152 coordinates (*x*, *y*, *z*)	Brain regions	*P*-value
Positive correlation, rsFC in NAc					
1	89	4.48	(10, −12, 46)	SMA	0.001
		4.04	(10, −16, 46)	Precentral gyrus	
		3.98	(8, −8, 42)	ACC	
		3.47	(12, −16, 50)	Precentral gyrus/unclassified White matter	
2	88	3.92	(−38, −28, 16)	Heschl's gyrus	0.001
		3.77	(−40, −28, 10)	Planum temporale	
		3.58	(−40, −32, 10)	Planum temporale	
		3.37	(−32, −28, 16)	Insula	
		3.18	(−46, 20, 14)	Central opercular cortex	

MNI, Montreal Neurological Institute; DARS, Dimensional Anhedonia Rating Scale; rsFC, resting-state functional connectivity; NAc, nucleus accumbens; SMA, supplementary motor area; ACC, anterior cingulate cortex.

a. Controlled for Montgomery–Åsberg Depression Rating Scale, gender and age.

### Independent component analyses

The independent component map representing the salience network was identified: the greatest regions of activation were located in the insula, dorsal ACC, amygdala, thalamus and substantia nigra pars compacta/VTA. In both analyses, no significant associations were found between change in DARS score and rsFC in regions of the salience network, when controlling for age and gender and after correcting for multiple comparisons.

## Discussion

Overall, our results demonstrate baseline and week 8 rsFC correlations between anhedonia improvement and adjunct aripiprazole treatment. Further, there was an improvement in anhedonia with adjunct aripiprazole, independent of change in depressive severity. Therefore, aripiprazole may be a promising treatment option for patients with MDD specifically experiencing anhedonia symptoms. As far as we are aware, this is the first study to report on the effectiveness of aripiprazole in treating anhedonia, and the brain regions associated with anhedonia improvement, in a cohort of participants with depression who had not responded to and 8-week trial of SSRI monotherapy.

Among those who received 8 weeks of escitalopram plus aripiprazole, stronger connectivity patterns between the ACC and PCC and ventral-posterior praecuneus were predictive of a decrease in anhedonia symptoms after 8 weeks of treatment. The PCC and praecuneus are two of the major regions implicated in the default mode network (DMN).^46^ There is evidence to support the presence of distinct anterior and posterior subregions within the praecuneus, each with different functional activation patterns. The posterior praecuneus plays a role in memory retrieval.^47^ The exact role of the PCC is not fully understood, but the majority of evidence suggests it is important for internally directed thought and cognitive control.^48^ Considering the dorsal ACC is a key node of the salience network (i.e. the brain network most consistently implicated in reward processing), these findings suggest that a stronger connection between the DMN and salience network may predict anhedonia improvement after aripiprazole treatment. Conversely, a reduced connectivity between the ACC and the anterior praecuneus was associated with improvement in anhedonia. The anterior praecuneus, which is part of the DMN, is important for self-centred mental imagery.^47^ It has been previously suggested in Menon's unifying triple network model that the relationship between the salience network and DMN is essential for attending to external stimuli, including rewarding stimuli, as the salience network suppresses DMN activity.^12,49^ The connectivity patterns between the salience network and DMN found in our current study, with both positive and negative correlations at specific regions of the DMN, support these previous findings and suggest a complex interplay between these two networks in reward processing.

Increased connectivity between the NAc and ventral-posterior praecuneus predicted anhedonia improvement. As previously stated, the posterior region of the praecuneus is involved in memory retrieval.^47^ Previous studies have found that effective processing and retrieval of reward-related memories are required for reward processing.^50^ Additionally, in the current analysis, increased connectivity between the NAc and the pons, cerebellum lobule IX and the splenium of the corpus collosum correlated with anhedonia response (although the corpus collosum is not generally explored by fMRI, as it is white matter, there is emerging evidence that fMRI findings in white matter are detectable and reliable^51^). These three brain regions are not classically implicated in anhedonia or reward processing, but they are necessary for the integration, processing and relay of neural signals in the brain. The raphe nuclei, where most serotonergic neurons originate, is located in the pons. Serotonin is involved in modulating dopamine activity in the NAc, and therefore plays a role in regulating reward response in the NAc.^52,53^ Lobule IX of the cerebellum is a ‘non-motor’ region of the cerebellum, implicated in emotional processing.^54^ Increasing evidence suggests the cerebellum may be part of the DMN,^55^ further supporting the theory that connectivity patterns between reward regions and the DMN is predictive of anhedonia improvement after aripiprazole. However, the cerebellum is not typically studied for its role in reward processing, so data supporting this finding are limited.

This study provides support for the role of dopamine in both reward-related processing and anhedonia treatment. To our knowledge, there have been no human studies assessing the effect of aripiprazole on anhedonia in a population with MDD. However, in preclinical studies, aripiprazole had favourable effects on animal models of anhedonia.^56,57^ Further, favourable effects of aripiprazole on anhedonia symptoms have also been reported in bipolar depression and schizophrenia.^58,59^ Aripiprazole likely has anti-anhedonia effects directly via partial D2 receptor agonism, and indirectly via 5-HT2A serotonin receptor antagonism,^59^ which increases dopaminergic activity by reducing blockade of dopamine receptors.^60^ In addition, aripiprazole is shown to increased dopaminergic activity in brain regions specifically implicated in reward processing, most notably the mesolimbic pathway.^61^ Aripiprazole's effect on dopamine is dose-dependent, with higher doses resulting in greater D2 receptor occupancy; aripiprazole occupies 74–85% of D2 receptors at doses of 2–10 mg (the dose range used in the current study).^62^

Interestingly, there was an inverse relationship between rsFC among key reward regions and decrease in anhedonia; that is, reduced connectivity at baseline between specific reward regions was associated with anhedonia improvement post-aripiprazole treatment. This may suggest that aripiprazole is more likely to act favourably on the subgroup of individuals with anhedonic brain-connectivity patterns, demonstrating that aripiprazole, and potentially other dopamine modulator agents, may be particularly effective in addressing reward-related deficits.

There were no significant findings in the ICA of the salience network, demonstrating no role of functional connectivity within the salience network in predicting anhedonia improvement. However, our current findings suggest that connectivity between regions of the salience network and other brain regions may predict anhedonia improvement after antidepressant treatment, considering our finding that that functional connectivity between the ACC (a major hub of the salience network) and regions of DMN was significantly associated with anhedonia improvement. This is supported by multiple other antidepressant biomarker studies reporting that connectivity between nodes of different brain networks is predictive of antidepressant response, but not connectivity within a network itself (reviewed by Dunlop et al^63^). However, the current study may be underpowered to detect relationships within the salience network, as ICA is data-driven, rather than model-driven, and therefore may not be suitable to detect more subtle associations in studies with smaller sample sizes.

In conclusion, eight weeks of aripiprazole, adjunct to escitalopram, was associated with improved anhedonia symptoms. Several distinct rsFC patterns were predictive of anhedonia improvement after treatment with adjunct aripiprazole. Decreased functional connectivity between key reward regions was associated with anhedonia improvement, suggesting that aripiprazole may be an effective treatment for individuals experiencing reward-related deficits. Future studies are required to replicate the present findings in larger placebo-controlled studies, and explore their generalisability with other agents with partial D2 agonism and/or 5HT2A antagonism.

### Limitations

Because only escitalopram non-responders received aripiprazole treatment, all participants in the aripiprazole treatment group were ‘treatment-resistant’ to at least one antidepressant. However, as aripiprazole is only approved as adjunct treatment for depression, when used in combination with a first-line antidepressant, this study cohort reflects a clinical group of individuals with depression who may be prescribed aripiprazole in clinical practice. Overall, the study population was moderately depressed and moderately anhedonic, and therefore results may not be applicable to individuals with more severe depression or anhedonia. Further, as presence of anhedonia was not an inclusion criterion, individuals without clinically significant anhedonia at baseline were part of the study cohort. This study was limited by the sample size, which may have been too small to detect associations, particularly in the ICA. Further, there was no placebo group to compare our findings against, and therefore we cannot make definitive conclusions about efficacy of aripiprazole in anhedonia or the specificity of resting-state biomarkers.

## Supporting information

Vaccarino et al. supplementary material Vaccarino et al. supplementary material

## Data Availability

The data that support the findings of this study are available from the corresponding author, S.H.K., on reasonable request.
