# Supplementary material for: Functional neuroimaging biomarkers of anhedonia response to escitalopram plus adjunct aripiprazole treatment for major depressive disorder
Source: BJPsych Open. 2024 Jan 5;10(1):e18. doi: 10.1192/bjo.2023.588 (PMC10790221; doi:10.1192/bjo.2023.588)
Supplement: Vaccarino et al. supplementary material [file S2056472423005884sup001.docx]

**Functional Neuroimaging Biomarkers of Anhedonia Response to Escitalopram Plus Adjunct Aripiprazole Treatment for Major Depressive Disorder**

**SUPPLEMENTARY MATERIAL**

**Resting-state functional MRI Association with Anhedonia Change After Escitalopram Monotherapy**

Introduction

For comparative purposes, in addition to the main analysis looking at fMRI biomarkers of anhedonia response to adjunct aripiprazole, a secondary analysis was completed to look for fMRI biomarkers of escitalopram monotherapy. Resting-state fMRI predictors of DARS change among individuals receiving escitalopram only were analyzed from (1) baseline to week 8 (i.e., the entire study population receiving initial escitalopram monotherapy) and (2) from baseline to week 16 (i.e., escitalopram responders who received escitalopram monotherapy throughout the entirety of the study).

Methods

All methods were the same as used in the main analysis.

Results

A total of n = 211 participants were enrolled; n = 134 of had complete clinical and neuroimaging data at baseline and week 8. n = 85 participants were escitalopram responders; n = 57 had complete data for baseline and week 16. Demographics are presented in Supplementary Table 1.

*I. Week 0-8, Seed-based correlation analysis: rsFC association with anhedonia change*

Change in DARS score among participants with MDD treated with eight weeks of escitalopram was positively correlated with rsFC at baseline between the ACC and the precentral gyrus (p = 0.004) after controlling for age, sex, and MADRS score. However, the region associated with the precentral gyrus was closely associated with unclassified white matter regions of the brain, only showing activity in a fraction of the entire grey matter region identified. Further, increase in DARS score was positively associated with rsFC between the NAc and the ACC (p = 0.006), supplementary motor area (p = 0.006), precuneus (p = 0.003), and intracalcarine cortex (p = 0.003) when controlling for age, sex, and MADRS score; specifically, significant associations within the precuneus and intracalcarine cortex were only significant when controlling for age, sex, and MADRS, but not age and sex alone. These results are presented in Supplementary Table 2.

*II. Week 0-16, Seed-based correlation analysis: rsFC association with anhedonia change*

There were no significant associations between change in DARS score over 16 weeks and baseline resting-state functional connectivity among individuals treated with 16 weeks of escitalopram only.

*Independent Component Analyses*

The independent component map representing the SN was identified: the greatest regions of activation were located in the insula, dorsal ACC, amygdala, thalamus, and SNc/VTA. In all analyses, no significant associations were found between change in DARS score and rsFC in regions of the SN, when controlling for age and sex and after correcting for multiple comparisons.

Discussion

Among MDD participants who received treatment with 8 weeks of escitalopram, baseline rsFC between the nucleus accumbens and (1) ACC and (2) precuneus were predictive of improvement in anhedonia symptoms. The functional connection between the NAc and ACC is essential for information regarding overall reward salience and motivation to be accurately integrated into higher-order cognitive processing and cost-benefit analysis (1,2). Our findings suggest that individuals with stronger functional connectivity between these key reward regions are more likely to report an improvement in anhedonia during escitalopram treatment. The precuneus is one of the main brain regions implicated in the default mode network (DMN), which is active during periods of internal mental-state processing, such as during self-directed thought and introspection (3,4). Based on our results, functional connectivity between the NAc and precuneus may promote reward responsivity, perhaps by switching cognitive activity away from internal state processing toward reward processing. It has been suggested in previous literature that decreased DMN activity is necessary for reward processing, so that attentional resources may be focused instead on external stimuli, rather than internal state processing (5). A previous study supports our findings, demonstrating that decreased connectivity between the NAc and DMN was associated with reward deficits among patients with MDD, as well as bipolar disorder, schizophrenia, and psychosis (6).

While some associations were found between functional connectivity in reward-related brain regions and anhedonia response, the findings were much less compelling than those found in the aripiprazole-treated group (see main text for details). Most of the regions associated with anhedonia response among escitalopram responders in the current analysis do not have a clear role in reward responsivity (i.e., SMA and intracalcarine cortex). Further, none of these findings were replicated in the 0-16 week analysis, and therefore may have been spurious findings. The findings of the relationship between the ACC and precuneus were also found in the aripiprazole treated group, and therefore this relationship may be more related to general antidepressant treatment and clinical recovery, rather than treatment with any specific medication.

References

1. Smith KS, Tindell AJ, Aldridge JW, Berridge KC. Ventral pallidum roles in reward and motivation. Behav Brain Res [Internet]. 2009 Jan;196(2):155–67. Available from: https://linkinghub.elsevier.com/retrieve/pii/S0166432808005391

2. Root DH, Melendez RI, Zaborszky L, Napier TC. The ventral pallidum: Subregion-specific functional anatomy and roles in motivated behaviors. Prog Neurobiol [Internet]. 2015 Jul;130:29–70. Available from: https://linkinghub.elsevier.com/retrieve/pii/S0301008215000271

3. Ekhtiari H, Nasseri P, Yavari F, Mokri A, Monterosso J. Neuroscience of drug craving for addiction medicine. In: Progress in Brain Research [Internet]. 2016. p. 115–41. Available from: https://linkinghub.elsevier.com/retrieve/pii/S0079612315001909

4. Gillespie CF, Szabo ST, Nemeroff CB. Unipolar depression. In: Rosenberg’s Molecular and Genetic Basis of Neurological and Psychiatric Disease [Internet]. Elsevier; 2020. p. 613–31. Available from: https://linkinghub.elsevier.com/retrieve/pii/B9780128138663000369

5. von Rhein D, Beckmann CF, Franke B, Oosterlaan J, Heslenfeld DJ, Hoekstra PJ, et al. Network-level assessment of reward-related activation in patients with ADHD and healthy individuals. Hum Brain Mapp [Internet]. 2017 May;38(5):2359–69. Available from: http://doi.wiley.com/10.1002/hbm.23522

6. Sharma A, Wolf DH, Ciric R, Kable JW, Moore TM, Vandekar SN, et al. Common dimensional reward deficits across mood and psychotic disorders: A connectome-wide association study. Am J Psychiatry [Internet]. 2017 Jul;174(7):657–66. Available from: http://ajp.psychiatryonline.org/doi/10.1176/appi.ajp.2016.16070774

Supplementary Table 1. Baseline Demographics of Study Population

| **Variable** | **Entire population**  **(n = 211)** | **Week 8 Responders**  **(n = 85)** | **Week 8 Non-responders**  **(n = 95)** | **p-value** |
| --- | --- | --- | --- | --- |
| Age (SD) | 35.3 (12.6) | 35.0 (12.2) | 35.8 (13.2) | 0.68 |
| Years of education (SD) | 16.9 (2.1) | 16.9 (2.3) | 16.9 (2.1) | 0.62 |
| % Female (n) | 63% (133) | 65% (55) | 60% (57) | 0.52 |
| % Employed (n) | 68% (131) ^a^ | 67% (52) | 67% (58) | 1.00 |
| % Never married (n) | 58% (122) | 62% (53) | 53% (50) | 0.19 |
| Ethnicity  % White (n)  % Asian (n)  % Latin/Hispanic (n)  % Black (n)  % Mixed (n)  % Other (n) | 70% (147)  15% (32)  5% (10)  3% (6)  6% (13)  1% (3) | 72% (61)  14% (12)  5% (4)  4% (3)  5% (4)  1% (1) | 69% (66)  16% (15)  4% (4)  1% (1)  7% (7)  2% (2) | 0.74  0.78  0.87  0.28  0.45  0.62 |
| Age of MDD onset (SD) | 20.9 (10.5) | 19.7 (9.2) | 21.9 (11.1) | 0.16 |
| Number of previous MDEs (SD) | 3.4 (4.4) | 3.5 (3.1) | 2.7 (3.4) | 0.11 |
| Current MDE duration, months (SD) | 25.8 (33.6) | 23.1 (29.5) | 30.5 (37.6) | 0.15 |
| Number of previous treatments (SD) | 0.57 (0.82) | 0.40 (0.68) | 0.72 (0.91) | **< 0.01** |
| % Family history, psychiatric illness (n) | 77% (160) ^b^ | 78% (64) | 75% (71) | 0.61 |
| Mean MADRS score (SD) | 29.9 (5.6) | 29.5 (5.6) | 30.5 (5.5) | 0.24 |
| Mean DARS score (SD), Baseline | 33.8 (14.2) | 34.0 (13.5) | 32.3 (15.1) | 0.43 |
| Mean DARS score (SD),  Week 8 | 41.1 (15.8) | 50.6 (12.0) | 32.6 (14.0) | **< 0.001** |
| Mean DARS score (SD), Week 16 | 46.7 (16.4) | 53.0 (11.8) | 41.2 (17.8) | **< 0.001** |
| Change in DARS (SD),  Baseline to Week 8 ^c^ | 1.50 (1.07) | 1.75 (1.00) | 1.28 (1.09) | **0.04** |
| Change in DARS (SD),  Baseline to Week 16 ^c^ | 1.72 (11.37) | 1.83 (1.14) | 1.62 (1.56) | 0.32 |
| Change in DARS (SD),  Week 8 to Week 16 ^c^ | 1.25 (0.51) | 1.10 (0.32) | 1.39 (0.61) | **< 0.001** |

^a^ n = 17 missing data, ^b^ n = 3 missing data, ^c^ expressed as a fraction, final value divided by initial; SD: standard deviation, MDD: Major Depressive Disorder, MDE: Major Depressive Episode, DARS: Dimensional Anhedonia Rating Scale; MADRS: Montgomery Asberg Depression Rating Scale

Supplementary Table 2. MNI152 coordinates of Z-maxima for association between DARS change and rsFC, baseline to week 8 ^a^

| **Cluster** | **# voxels** | **Z-max** | **MNI152 coordinate(s) [x, y, z]** | **Brain region(s)** | **p** |
| --- | --- | --- | --- | --- | --- |
| **Positive correlation, rsFC in ACC** | | | | | |
| 1 | 147 | 6.47 | [14, -20, 48] | Precentral gyrus/unclassified WM | 0.005 |
| **Positive correlation, rsFC in NAc** | | | | | |
| 1 | 108 | 4.49  3.75  3.56  3.36 | [-22, -60, 4]  [-12, -76, 14]  [-18, -74, 6]  [-16, -74, 10] | Precuneus  Intracalcarine cortex  Intracalcarine cortex  Intracalcarine cortex | 0.003 |
| 2 | 82 | 4.31  3.88  3.86 | [12, -16, 50]  [6, -6, 42]  [10, -12, 46] | Precentral gyrus/unclassified WM  ACC  SMA | 0.013 |

^a^ Controlled for age, sex, and MADRS; MNI: Montreal Neurological Institute; DARS: Dimensional Anhedonia Rating Scale; rsFC: resting-state functional connectivity; WM: white matter; ACC: anterior cingulate cortex; SMA: supplementary motor area
